# Non-Native Plant Litter Enhances Soil Carbon Dioxide Emissions in an Invaded Annual Grassland

**DOI:** 10.1371/journal.pone.0092301

**Published:** 2014-03-19

**Authors:** Ling Zhang, Hong Wang, Jianwen Zou, William E. Rogers, Evan Siemann

**Affiliations:** 1 College of Resources and Environmental Sciences, Nanjing Agricultural University, Nanjing, China; 2 Department of Ecology and Evolutionary Biology, Rice University, Houston, Texas, United States of America; 3 Department of Horticulture, Cornell University, Ithaca, New York, United States of America; 4 Department of Ecosystem Science and Management, Texas A & M University, College Station, Texas, United States of America; Helmholtz Centre for Environmental Research – UFZ, Germany

## Abstract

Litter decomposition is a fundamental ecosystem process in which breakdown and decay of plant detritus releases carbon and nutrients. Invasive exotic plants may produce litter that differs from native plant litter in quality and quantity. Such differences may impact litter decomposition and soil respiration in ways that depend on whether exotic and native plant litters decompose in mixtures. However, few field experiments have examined how exotic plants affect soil respiration via litter decomposition. Here, we conducted an *in situ* study of litter decomposition of an annual native grass (*Eragrostis pilosa*), a perennial exotic forb (*Alternanthera philoxeroides*), and their mixtures in an annual grassland in China to examine potential invasion effects on soil respiration. *Alternanthera* litter decomposed faster than *Eragrostis* litter when each was incubated separately. Mass loss in litter mixes was more rapid than predicted from rates in single species bags (only 35% of predicted mass remained at 8 months) showing synergistic effects. Notably, exotic plant litter decomposition rate was unchanged but native plant litter decomposition rate was accelerated in mixtures (decay constant *k* = 0.20 month^−1^) compared to in isolation (*k* = 0.10 month^−1^). On average, every litter type increased soil respiration compared to bare soil from which litter was removed. However, the increases were larger for mixed litter (1.82 times) than for *Alternanthera* litter (1.58 times) or *Eragrostis* litter (1.30 times). Carbon released as CO_2_ relative to litter carbon input was also higher for mixed litter (3.34) than for *Alternathera* litter (2.29) or *Eragrostis* litter (1.19). Our results indicated that exotic *Alternanthera* produces rapidly decomposing litter which also accelerates the decomposition of native plant litter in litter mixtures and enhances soil respiration rates. Thus, this exotic invasive plant species will likely accelerate carbon cycling and increase soil respiration even at intermediate stages of invasion in these annual grasslands.

## Introduction

Litter decomposition has been shown to be an important ecosystem process associated with carbon (C) cycling and nutrient release [1], [2]. Exotic plant invasions can enhance plant litter decomposition and C cycling of invaded ecosystems [3]–[5]. During decomposition, more C may go belowground as substrate for soil microbial activities [6]. In addition, invasive plant species usually have larger net primary production (NPP) relative to the native species via higher C fixing efficiency [3], [7]. Thus, exotic plant invasions are commonly accompanied by higher litter inputs and more C input belowground.

Native ecosystems are generally encroached or invaded over a prolonged time period. Consequently, invasive plant litter mixes with litter of native species and may not be decomposing alone over the course of a typical invasion. Mixing invasive plant litter with native plant litter of different quality may enhance decomposition of the litter mixture via nutrient transfers between component plant litters, leading to non-additive effects [8]–[13]. Thus, enhanced decomposition rates of litter mixtures containing both native and invasive plant detritus may be common in invaded ecosystems, even though results might vary among different invasions [14]. Alterations of litter decomposition and C cycling following exotic plant invasions may be underestimated based on assessments of decomposition using litter of only a single species [9], [11]. Consequently, empirical studies measuring the effects of litter mixing will help to better predict the effects of plant invasions on nutrient dynamics.

Litter decomposition processes involve C transfers between plant litter and the soil ecosystem at the litter-soil interface [6], [15]. Carbon cycling during litter decomposition includes C loss to the atmosphere as CO_2_ emissions and input to belowground organic pools [6]. However, while higher litter decomposition rates may lead to more C input belowground, this does not necessarily mean equivalent C sequestration in soil [6], [16], [17]. In general, soil C stocks are a balance of photosynthetic C input and output via CO_2_ emissions [18]. Hence, CO_2_ emissions at the soil-atmosphere interface play an important role in C loss from terrestrial C pools [19], [20]. These emissions have been estimated to be the largest source of CO_2_ released to the atmosphere, most notably being an order of magnitude larger than the combined sum of C emissions by anthropogenic fossil fuel combustion and deforestation [20]. Thus, even a slight shift in soil CO_2_ emissions may lead to large changes in atmospheric composition and increase rates of global climate change. Carbon released belowground by accelerated litter decomposition may provide fresh C substrate for soil microbes to decompose but may also enhance decomposition of recalcitrant forms of C. This can lead to high levels of additional CO_2_ release through this positive priming effect [6], [21]–[23]. However, few studies have been conducted *in situ* to examine how soil CO_2_ emissions are affected by litter composition [24], [25], especially native ecosystems that are experiencing exotic plant encroachment or invasions.

*Eragrostis pilosa* (Poaceae, Indian love grass, hereafter referred to as “*Eragrostis*”) is a native annual grass in China with a wide global distribution [26]. The grassland dominated by *Eragrostis* in our experimental site is being invaded by the perennial exotic forb *Alternanthera philoxeroides* (Amaranthaceae, alligator weed, hereafter referred to as “*Alternanthera*”) introduced from South America via Japan [27]. Both plant species occurred in this invaded grassland and have coexisted for ∼5 years. During the invasion by *Alternanthera*, litter produced by the native and the invasive plant have been decomposing both alone and in mixture. While a number of studies have been conducted to examine native and invasive plant litter decomposition individually, few studies have tried to examine litter mixing effects on litter decomposition and soil respiration rates in invaded areas. Here, we conducted an *in situ* experiment to examine the effect of litter addition and litter mixing on soil CO_2_ emissions using the native plant *Eragrostis* and invasive plant *Alternanthera* as model species. By examining decomposition rates of single- and mixed-species litter, as well as CO_2_ emissions from soils with different litter compositions (i.e., single-species litters, mixed-species litter, and litter-free controls), we addressed the following questions: 1) Do native and invasive plant litter differ in litter quality and decomposition rates? 2) Does litter mixing have non-additive effects on decomposition; 3) Do CO_2_ emissions from litter-soil systems vary between litter types and species?

## Materials and Methods

### Study Site

The experiment was conducted in 2010–2011 in an unmanaged annual grassland at the Nanjing Agricultural University experimental station (6 m elevation, 32°01′N 118°37′E) in China. Mean monthly temperatures in this area range from −10°C in January to 32.5°C in July with a mean annual temperature of 25°C. Annual mean precipitation of this area is 980 mm with up to 90% of precipitation occurring between March and November. Soils are classified as hydromorphic soils in a Chinese soil taxonomic classification and are high in clay content (6% sand, 40% silt and 54% clay) with an initial pH (H_2_O) of 6.8 and organic carbon content of 8.3 kg C m^−2^.

### Focal Species

*Eragrostis pilosa* is an annual bunchgrass native to China and is distributed throughout southeast China. The field station was dominated by naturally occurring *Eragrostis* before the introduction of exotic invasive plants. Presently, remnants of the native *Eragrostis* dominated grassland are threatened by *Alternanthera* which is expanding its range. The co-existence of *Eragrostis* with this invasive species and its broad distribution make *Eragrostis* a useful model plant to study ecosystem C cycling changes after exotic plant invasions.

The most abundant invasive plant at the field station is the perennial forb *Alternanthera philoxeroides* that covers more than 50% of the grassland during peak growing time. *Alternanthera* originates from South America, was introduced to China via Japan in the 1930s, and has been reported to be invasive in at least 19 provinces [27]. Moreover, it is also invasive in Australia and North America. Basset et al. [28] reported faster litter decomposition rates of invasive *Alternanthera* in New Zealand, indicating it may potentially facilitate ecosystem element cycling in areas it invades.

### Litter Decomposition

An *in situ* litterbag approach was used to test the litter decomposition rate of both focal species. At the end of the 2010 growing season, we hand collected newly produced litter from standing *Eragrostis* and *Alternanthera* at the Nanjing Agricultural University experimental station. Neither plant species is endangered or protected. All litter samples were air dried and carefully processed (cut into a size of litter length at 3∼6 cm) to fit into the litterbag. For each litter treatment, 10 g of litter was put into 10×15 cm litterbags (mesh size of 1 mm^2^) [29], [30]. Single species bags received 10 g of a single litter type. For the mixed-species treatment, 5 g of each litter type was put in the bag. All litterbags were deployed simultaneously in invaded areas where both species co-existed in March 2011 and were retrieved after *in situ* decomposition for 3, 6 or 8 months. A subsample of litter of each species was oven dried for water, C and N measurement. Samples used for chemical properties measurement were ground to pass through a 2 mm sieve, decarbonized with HCl, and then analyzed for C and N content by a CNS elemental analyzer (Variomax CNS Analyzer, Elementar GmbH, Hanau, Germany) [31].

### *In situ* soil CO_2_ emission measurements

In the invaded areas where both plants coexisted, 12 plots (2×2 m and 5 m apart) were set up in December 2010, prior to the 2011 growing season by thoroughly removing all existing plant materials. The shallow root depth of both plant species enabled the thorough clearance of belowground biomass. Soil CO_2_ emissions were measured weekly for 8 months from plots that had existing litter removed and then received different litter compositions (i.e. litter of *Alternanthera*, *Eragrostis* or mixtures of both plant) and from blank control plots with litter removed and no subsequent litter addition. CO_2_ was measured with the static-opaque chamber method, a commonly used, well-established method [32]. Within each plot, circular grooved clay collars (height = 25 cm, inside diameter = 20 cm) were installed flush with the soil surface for permanent use. The area within the clay collars received litter treatments of 10 g litter of *Alternanthera*, *Eragrostis* or 1∶1 mixture. Mesh cloth was placed above the clay collars to prevent new litterfall and to fix the litterbags to the ground. While soil CO_2_ emissions were measured, an open-bottomed cylindrical PVC gas sampling chamber (100 cm high) was fit into the circular groove. Each groove was filled with water to seal the rim of the opaque cylindrical chamber. The gas sampling chambers were wrapped by foam and aluminum foil to minimize temperature variations during measurements. Battery-powered fans mixed the air inside the chambers. Three samples of headspace gas were collected using single-use syringes. The first sample was collected when chambers were installed (0 minutes) followed by collections 10 and 20 minutes after installation. A gas chromatograph (Agilent 7890) with a flame ionization detector (FID) was used to measure CO_2_ concentrations. Soil CO_2_ emission rates were determined from the slope of the mixing ratio change of the three samples using the following equation: 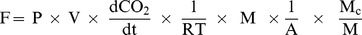
where *F* refers to rate of soil CO_2_ emission rate (mg CO_2_–C m^−2^ h^−1^), *P* is the standard atmospheric pressure (Pa), *V* and *A* are the volume (m^3^) and interior bottom area (m^2^) of the cylindrical chamber, *R* stands for universal gas constant, *T* is the absolute air temperature (K) when the gas sample was aspirated and *Mc* and *M* are the molecular masses of carbon and CO_2_ (g mol^−1^), respectively. Cumulative CO_2_ emissions within a given time period were calculated by multiplying average CO_2_ emission rates and the associated time span assuming that measurements were representative. Since the C content of the plant litter differed, cumulative CO_2_ emissions are also reported normalized to litter C input (g CO_2_-C per g litter-C input) and emissions from bare soil were subtracted when discussing effects of the different litter quality on CO_2_ emissions.

### Statistics

Effects of litter mixing and decomposition time on *Alternanthera* and *Eragrostis* litter mass were tested by two-way analysis of variance (ANOVA). Two-way ANOVA was also used to test the effects of plant species and decomposition time on litter mass remaining within each litter type (in isolation or in mixtures). Post-hoc student's *t* tests were used to examine significant differences among treatment means for factors with more than 2 levels.

Litter decay constants (*k*, month^−1^) were calculated to examine litter mixing effects on litter decomposition rates. The *k* values were calculated from a single negative exponential model as following [33], [34]:
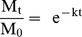
where *M_t_* is litter mass remaining at time *t* and *M_0_* is initial litter biomass at time *t* = 0, and *k* is a first-order litter decay constant. The mean *k* values of litter decomposing in isolation and in mixture of both species were also calculated by the model. Litter mass remaining was examined using all combinations of the three single-species or mixed-species results at each time point (9 possible combinations).

Effects of litter mixing on overall litter decomposition rates were examined by comparing observed mass remaining in mixed-species bags and expected mass remaining of litter mixtures. Expected mass remaining was obtained based on decomposition rate of single plant litter samples according to the following equation [35], [36]:
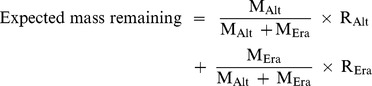
where *R* refers to the remaining litter mass of a species (*Alt*  =  *Alternathera*, *Era*  =  *Eragrostis*) in the single-species litterbag and *M* refers to the initial litter mass of a species in the litter mixture. A randomization approach was used in calculating expected mass remaining. Specifically, the set of all combinations of the three single-species results of each species (9 possible combinations) were used to generate expected mass remaining. Post-hoc student's *t* tests were used to examine differences between observed and expected litter mass remaining at each time point.

Statistical results at *α* = 0.05 were considered significant. All statistical analysis was conducted in JMP 9.0 (SAS Inc., Cary, NC, USA).

## Results

### Litter Chemistry and Litter Mass Loss

**Figure 1:**
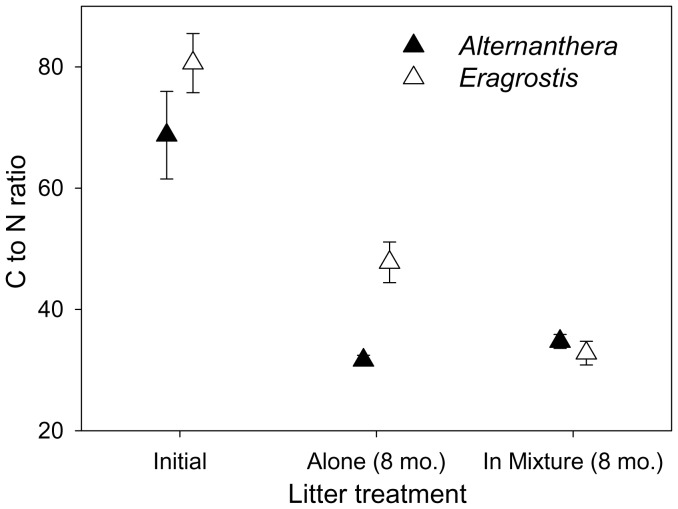
Mean initial and final C:N ratio of *Alternanthera* and *Eragrostis* litter decomposing both alone and in mixture. Means ±1 SE are shown.

Initial litter quality of invasive *Alternanthera* (68.74±5.90) did not differ from that of native *Eragrostis* (80.63±3.99; *F*_1, 4_ = 2.79, *P* = 0.1702; Fig. 1) in terms of C:N ratio, but initial N concentration of *Alternanthera* litter (9.16±0.27 g kg^−1^) was significantly higher than that of *Eragrostis* litter (6.69±0.08 g kg^−1^; *F*_1, 4_ = 15.73, *P* = 0.0166). However, after 8 months of decomposition in single species bags, litter C:N ratio of *Alternanthera* (31.63±0.66) was significantly lower than that of *Eragrostis* (47.76±2.73; *F*_1, 4_ = 32.95, *P* = 0.0046) and was similar after 8 months of decomposition in mixture (Fig. 1).

**Figure 2:**
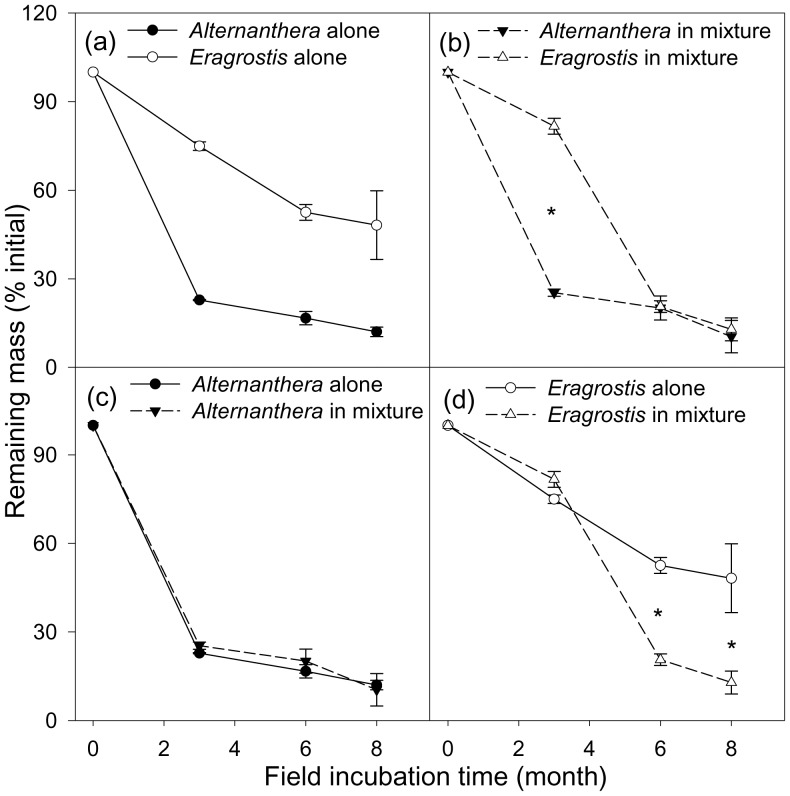
Remaining mass (% initial) of native and invasive plant litter when each of them was decomposing alone (a) or in mixtures (b). Dynamics of litter mass remaining between single-species and litter mixtures within *Alternanthera* and *Eragrostis* were presented in (c) and (d), respectively. Asterisks indicate time points when means were significantly different at α = 0.05.

When each was decomposing alone, *Alternanthera* litter decomposed faster than *Eragrostis* litter (Table 1; Fig. 2a). The litter decay constant (*k*) of *Alternanthera* was approximately three times that of *Eragrostis* when each was decomposing alone (Table 2). Litter mass loss rate of *Eragrostis* was significantly accelerated by litter mixing (Table 1; Fig. 2cd) with *k* values doubled but mixing did not change the mass loss rate of *Alternanthera* litter (Table 2). This decreased the difference in mass loss rate between the two species in mixtures (Fig. 2b). Synergistic non-additive effects on litter mixture decomposition were observed after 6 and 8 months of decomposition (Table 3).

**Table 1:** ANOVAs for remaining litter mass as affected by species and litter mixing with time in the field incubation study.

		Single species		Mixed species	
Factor	*df*	*F value*	*P value*	*F value*	*P value*
Species	1,12	**128.1**	**<0.0001**	**70.9**	**<0.0001**
Time	2,12	**9.0**	**0.0049**	**118.0**	**<0.0001**
Interaction	2,12	**2.0**	**0.1804**	**60.8**	**<0.0001**
Model	5,12	**32.0**	**<0.0001**	**85.7**	**<0.0001**

		*Alternanthera*		*Eragrostis*	
Factor	*df*	*F value*	*P value*	*F value*	*P value*
Litter mixing	1,12	0.4	0.5219	**32.0**	**0.0001**
Time	2,12	**11.1**	**0.0023**	**71.2**	**<0.0001**
Interaction	2,12	0.5	0.6034	**14.3**	**0.0007**
Model	5,12	**5.0**	**0.0127**	**40.6**	**<0.0001**

Significant results are shown in bold.

**Table 2:** Litter mass decay constants (*k*, month^-1^) during litter decomposition in single- (*k_alone_*) or mixed-species litterbags (*k_mixed_*) after 8 months of decomposition in the field.

	*K_alone_*					*K_mixed_*				
Species	Estimate	S.E.	*t* ratio	*P* value	*R^2^*	Estimate	S.E.	*t* ratio	*P* value	*R^2^*
*Alternanthera*	0.37	0.04	9.45	<0.0001	0.97	0.36	0.04	9.12	<0.0001	0.95
*Eragrostis*	0.10	0.01	8.67	<0.0001	0.89	0.20	0.03	6.00	0.0001	0.86
Mean	0.18	0.01	21.59	<0.0001	0.95	0.24	0.01	19.65	<0.0001	0.98

Values were calculated from a first order negative exponential model.

**Table 3:** Expected and observed litter mass remaining (g) in mixed-species litter bags after field incubation for 3, 6 and 8 months.

	Litter mass remaining (g)			
Time	Expected	Observed	*t* ratio	*P* value
**3 months**	5.99±0.46	5.38±0.07	1.39	0.2028
**6 months**	3.30±0.05	2.08±0.10	**9.23**	**<0.0001**
**8 months**	3.33±0.25	1.17±0.14	**6.83**	**0.0001**

Predicted remaining mass was calculated from litter mass measured in single-species litter bags. Means ± SE. Differences between predicted and observed values were examined by Student's *t* tests. Significant results are shown in bold.

### Soil CO_2_ Emissions

**Figure 3:**
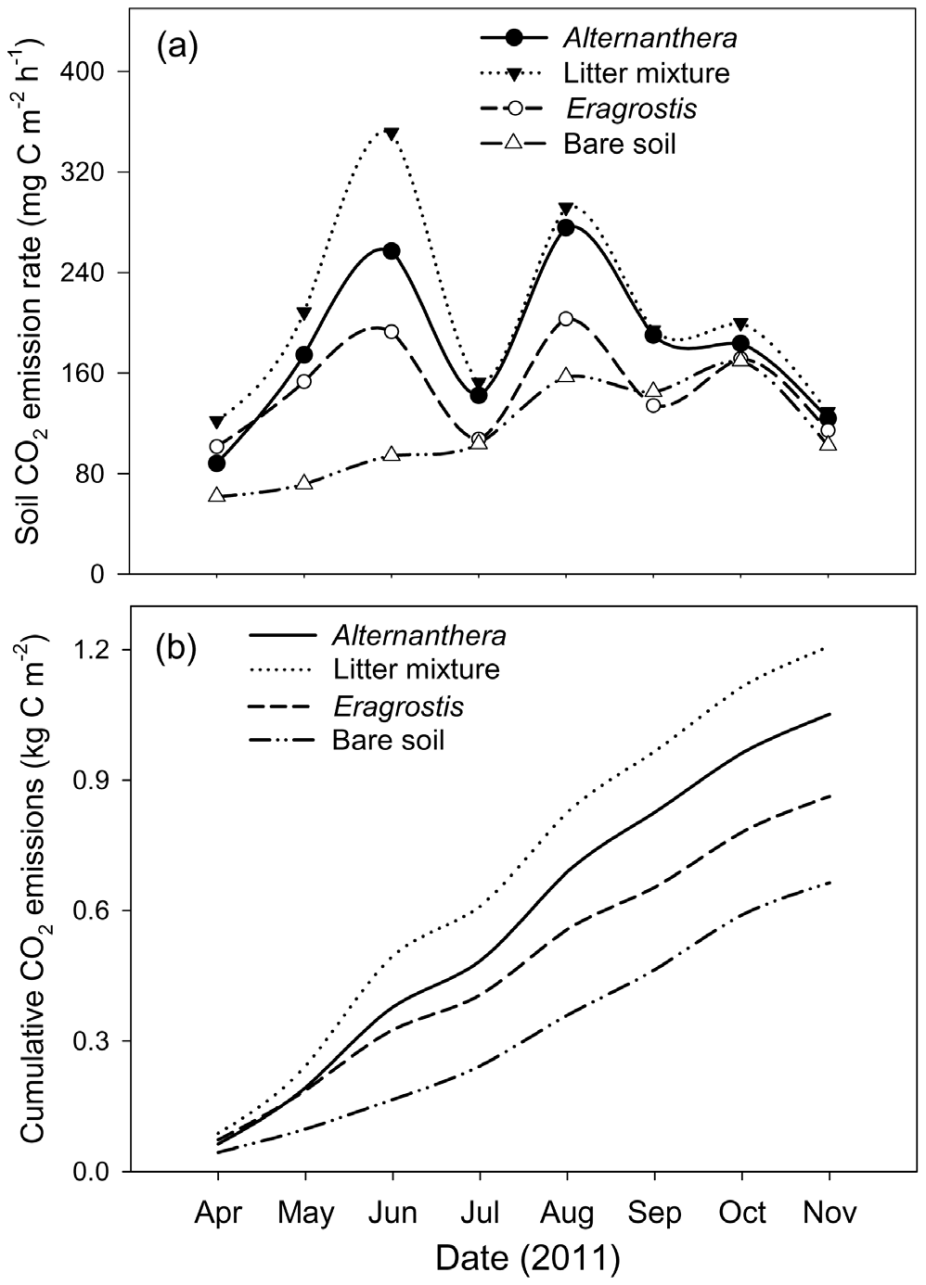
Monthly mean soil CO_2_ emission rates based on weekly measurements (a) and accumulated soil CO_2_ emissions (b) of soils with different litter types and soils without litter coverage (bare soil).

CO_2_ emission rates were highest from soils covered by litter mixtures, followed by soils with only *Alternanthera* litter and only *Eragrostis* litter, respectively (Fig. 3a). Soils with all litter removed (bare soil) were characterized by the lowest CO_2_ emission rates (Fig. 3a). Finally, cumulative CO_2_ emissions were consistent with the CO_2_ emission rates of soils with different litter coverage (Fig. 3b). The net increases in cumulative soil CO_2_ emissions from soils covered with litter compared to bare soils were 544 g CO_2_–C m^−2^ for mixtures, 388 g CO_2_–C m^−2^ for *Alternathera* litter, and 199 g CO_2_–C m^−2^ for *Eragrostis* litter. Net increases in carbon released as CO_2_ relative to carbon input as litter were also higher for mixed litter (3.34 g CO_2_–C per g litter-C) than for *Alternathera* litter (2.29) or *Eragrostis* litter (1.19).

## Discussion

Litter decomposition is an important ecosystem process releasing C and nutrients bound up in plant litter. Thus, changes in litter decomposition might profoundly influence ecosystem C cycling. In fact, as evidenced by our study, litter of the invasive plant *Alternanthera* had higher initial N concentration and decomposed faster compared to that of native *Eragrostis* when each species decomposed in isolation. Moreover, mixing the litter of both native and invasive species significantly enhanced litter mass loss rate of *Eragrostis*. Observed litter mass remaining in mixtures was lower than expected based on single species results, indicating synergistic non-additive effects of litter mixing on litter mixture decomposition. In turn, both CO_2_ emission rate and cumulative CO_2_ emissions were higher from soils under litter mixtures composed of both native and invasive plant litter relative to those with a single litter type, especially *Eragrostis*, or bare soils, thereby indicating that the faster decomposition rate of invasive plant litter and accelerated decomposition rate of litter mixtures can substantially contribute to higher soil CO_2_ emissions.

### Faster Decomposition Rate of Invasive Plant Litter

Litter of the invasive plant *Alternanthera* had higher N concentration and a faster decomposition rate which was consistent with other studies of invasive plants [3], [4], [28]. In general, the contact area of decomposing litter with soil could be an important factor enhancing litter decomposition via effects on environmental conditions such as soil water content and soil microbial communities. In this study, the litter samples were processed to be similar length and size and litterbags were fixed to the ground by mesh cloth. Therefore, the differences in litter contact area with soil were likely minimal. Along with environmental conditions, litter quality has been considered an important factor controlling litter decomposition rate. Indeed, litter of the invasive plant *Alternanthera* had higher N concentration and lower initial C to N ratio compared to *Eragrostis* litter (Fig. 1), indicating high quality litter for microbes to metabolize. Since invasive plant species may have greater ability to uptake soil nutrients than native plants [37], in some cases, higher soil nutrient uptake may lead to higher litter quality for the invasive plant [7]. *Alternanthera* litter was reported to decompose faster than litter from native sedge species (*Schoenoplectus tabernaemontani, Isolepis prolifer*) in a litterbag experiment in New Zealand [28]. However, in our study, the dominant native plant *Eragrostis* produced litter with lower N concentration relative to that of the invasive *Alternanthera* and hence had a slower mass loss rate. Consequently, there may be effects of *Alternanthera* invasions on litter decomposition and element cycling both through displacement of native species but also through litter mixing during the invasion process.

### Synergistic Effects of Litter Mixing on Litter Mixture Decomposition

Recent studies have shown that effects of litter mixing on decomposition may be non-additive when there is variation in component litter properties [8], [11], [12], [38]. In this study, synergistic non-additive effects of litter mixing on litter mixture mass loss were also observed (Table 3; Fig. 2) [11], [24], [39]. Decomposition of the lower quality *Eragrostis* litter was accelerated by mixing with *Alternanthera* litter (Table 2; Fig. 2). Moreover, mass loss rate of litter mixture was faster after 6 and 8 months of decomposition than expected based on each litter type decomposing alone (Table 3). Several mechanisms have been proposed to explain effects of litter mixing including microclimate conditions, secondary chemical release, and nutrient transfer [40]–[42]. In this study, litterbags were deployed among similar plant and soil environmental conditions, so it is likely that variations in microclimate were minimal. Moreover, even though a recent study reported allelopathic effects of *Alternanthera* on algal growth [43], releasing of secondary compounds would have been expected to reduce the rate of decomposition of *Eragrostis*, not accelerate it (Tables 1 and 2; Fig. 2) [41]. Thus, nutrient transfer may be the most likely cause of synergistic effects of litter mixing in this study [8], [9]. When decomposed in isolation, *Alternanthera* litter had significantly lower C to N ratio than *Eragrostis* litter after 8 months. When decomposed in mixture, however, both had similar C to N ratio, indicating potential N transfer between component species (Fig. 1). Together these results suggest that nutrient transfer likely caused the non-additive effects of litter mixing for these two species [8], [11].

### Higher Soil CO_2_ Emissions as Affected by Litter Addition

Soil CO_2_ emissions were enhanced by both invasive and native plant litter additions, especially when litter was a mixture of both component species (Fig. 3). Isotope labeling studies have shown that C input belowground is often the major C flux associated with litter biomass loss [6] so it is possible to have rapidly decomposing litter that increases soil C but not necessarily CO_2_ emissions. Here, *Eragrostis* litter had low rates of mass loss and low rates of soil CO_2_ emissions. The mass loss of *Alternanthera* and the litter mixtures were each much larger (Fig. 2). Moreover, with emissions from bare soils subtracted, soil CO_2_ emissions were much higher for the mixtures compared to either *Alternanthera* litter or *Eragrostis* litter added alone (Fig. 3). However, litter mixing that had synergistic non-additive effects on litter mass loss may lead to more C loss as CO_2_ emissions. Indeed, a similar result was reported by a study on the combined effect of beech-spruce litter on CO_2_ emissions. The highest CO_2_ emissions were found from soils covered by litter mixtures that had faster litter mass loss rate, even though results varied between bedrocks [24].Therefore, the invasion process of *Alternanthera* might be associated with higher soil CO_2_ emissions due to faster litter decomposition. In addition, invasive plant species often have greater C fixing capacity relative to the native species [7]. In another study at this research site, we found *Alternanthera* produced 1.6 times as much aboveground litter as native *Eragrostis* (data in review). Thus, in *Alternanthera* invaded areas, soil CO_2_ emissions and belowground C input may increase due to both greater litter production as well as higher mass specific input [23] with the degree of mixing influencing the relative magnitude of these two effects [16]. More studies considering both aboveground and belowground litter decomposition would help to obtain a thorough understanding of exotic plant invasion effects on soil C dynamics.

### Litter Effects on Invasions

Differences in litter composition and decomposition may influence the process of invasion [4], [44]. High quality, rapidly decomposing litter may indirectly enhance the survival and performance of invasive species by elevating soil nutrient availability [4], [45], [46]. On the other hand, a recent study on invasive plant *Phragmites australis* in New England wetlands reported positive litter legacies on competitive ability of its offspring through buildup of slow decomposing litter that suppressed native plant recruitment [44]. Even though litter legacy effects on offspring performance were not examined in this study, the high nitrogen litter of *Alternanthera* suggests that positive effects through increasing soil nitrogen may occur [23].

## Conclusions

Our study showed that the invasive plant *Alternanthera philoxeroides* produced litter with faster decomposition rate than the dominant native species, *Eragrostis pilosa*. Mixing litter of invasive *Alternanthera* with that of native *Eragrostis* leads to synergistic non-additive effects on litter mixture decomposition and soil CO_2_ emissions. Differences in litter quality and nutrient transfers between component species might be factors contributing to the non-additive effects. Soil CO_2_ flux of areas with both native and invasive plant litter was higher than areas with equivalent invasive plant litter, native plant litter, and litter removal. The accelerated litter decomposition rate and enhanced soil CO_2_ emissions following invasion have implications for C sequestration in invaded ecosystems and the potential range expansion of invasive plant. The effects of litter mixing suggest that these effects may depend on the spatial scale and extent of incomplete or ongoing invasions. It should be noted that our study included only one native and one invasive species that co-occurred in an invaded ecosystem and may not characterize other invasions. More studies that investigate additional invasive plant species effects on ecosystem processes should be conducted to generalize our results to other kind of invasions associated with various species or invaded ecosystems.
